# Association between family support and 6-months functional recovery in acute ischemic stroke patients: a prospective cohort study

**DOI:** 10.3389/fmed.2025.1684785

**Published:** 2025-12-17

**Authors:** Ming Zhong, Yiheng Zhou, Yu Jia, Yi Yao, Yu Cheng, Dongze Li, Yonggang Zhang, Yi Lei, Qian Zhao, Jiajun Huang, Xiaoyang Liao

**Affiliations:** 1 Department of Neurology, The Second People’s Hospital of Neijiang, Neijiang, Sichuan, China; 2 General Practice Ward/International Medical Center Ward, Teaching and Research Section of General Practice, General Practice Medical Center, West China Hospital, General Practice Research Institute, Sichuan University, Chengdu, China; 3 Department of Emergency Medicine, Institute of Disaster Medicine and Institute of Emergency Medicine, West China Hospital, Sichuan University, Chengdu, China; 4 Nursing Key Laboratory of Sichuan Province, Department of Periodical Press, National Clinical Research Center for Geriatrics, Chinese Evidence-based Medicine Center, West China Hospital, Sichuan University, Chengdu, China

**Keywords:** acute ischemic stroke, family support, functional recovery, medication adherence, cohort study

## Abstract

**Aims:**

Stroke is a leading cause of disability and death worldwide, with family support playing a pivotal role in the recovery process. This study aimed to evaluate the association between family support levels and the short-term functional prognosis of patients with acute ischemic stroke.

**Materials and methods:**

A total of 124 patients admitted to the Department of Neurology at Second People’s Hospital of Neijiang were included, with an average age of 68 years. Family support was assessed by the Family Support Questionnaire (FSQ) and the Family APGAR Questionnaire. The primary outcome was functional independence, defined as a Modified Rankin Scale (mRS) score of 0–2, assessed 6 months after discharge. Secondary outcomes included lifestyle behaviors and medication adherence. Multivariate logistic and linear regression were used to analyze the association between family support and functional independence.

**Results:**

Compared to patients with FSQ of 0–5, FSQ of 10–15 were significantly associated with greater functional independence (OR: 1.666, 95% CI 1.236–2.214, *P* = 0.005) and mRS changes between baseline and follow-up at 6-months (β = −1.001, 95% CI −1.418 to −0.584, *P* < 0.001). However, the APGAR score was not significantly associated with functional recovery (*P* > 0.05). Lifestyle improvements were noted post-stroke, but no significant differences were observed among different family support levels (*P* > 0.05). Higher FSQ scores and APGAR scores were associated with better medication adherence (*P* < 0.001).

**Conclusion:**

The study concludes family support is positively associated with functional recovery and medication adherence, further studies are needed to clarify whether improved medication adherence mediates this association.

## Introduction

Stroke remains a global public health challenge, causing a substantial burden on socioeconomic development and human health (1–4). As the second leading cause of death worldwide, stroke-related mortality has exhibited significant growth over the past three decades (1, 2). Notably, ischemic stroke dominates among stroke subtypes, with both its incidence and disability rates demonstrating persistent upward trends (5). While thrombolysis and thrombectomy therapies have proven effective for acute ischemic stroke, limitations in treatment time windows and healthcare accessibility result in fewer than 1% of patients receiving timely intervention, with over half of survivors developing severe functional impairments (6–10). Against the backdrop of population aging-marked by multi-organ functional decline and comorbidities in elderly patients-post-stroke recovery becomes increasingly challenging (11), adding to the socioeconomic burden that needs to be addressed.

Families, as foundational societal units, play an irreplaceable role in stroke rehabilitation (12). Family support encompasses material assistance, emotional care, financial aid, and social resource coordination (13). By addressing daily living needs, enhancing treatment adherence, and facilitating rehabilitation exercises, such support positively impacts post-stroke recovery and stroke recurrence prevention (14, 15). However, the benefits of family support for the recovery of stroke patients are still in dispute. Some studies found that family support may be related to the psychological recovery of stroke patients, but not to their physical wellbeing (16, 17). This may be related to patients having received good rehabilitation treatment during their hospital stay. In fact, patients’ income and economic level are independent factors affecting the recovery of stroke patients, which may influence access to medical resources (18, 19). In resource-poor areas, family support is particularly important in the rehabilitation of stroke patients (20). Nevertheless, chronic diseases may diminish the family support available to patients, thereby compromising their recovery (21). Therefore, whether family support for stroke patients in resource-poor areas has a positive effect on their recovery is still unknown.

Since post-stroke dysfunction is the leading cause of disability in China (22, 23), rehabilitation interventions are necessary. Functional recovery refers to the degree of recovery of body structure and function to the pre-stroke state, which mainly occurs in the first 6 months after stroke, especially in the first 3 months (24–26). However, in China, due to the imperfection of primary healthcare institutions and high hospitalization costs, the rehabilitation of stroke patients primarily takes place at home (27, 28). Therefore, it is necessary to evaluate the short-term functional recovery of patients with acute ischemic stroke with different family support levels, and understand the prognosis of short-term functional recovery of patients with acute ischemic stroke with different family support levels. This study aims to clarify how distinct family support level influence short-term functional prognoses in acute ischemic stroke patients, thereby providing a theoretical basis for home-based rehabilitation strategies.

### Population and study design

**FIGURE 1:**
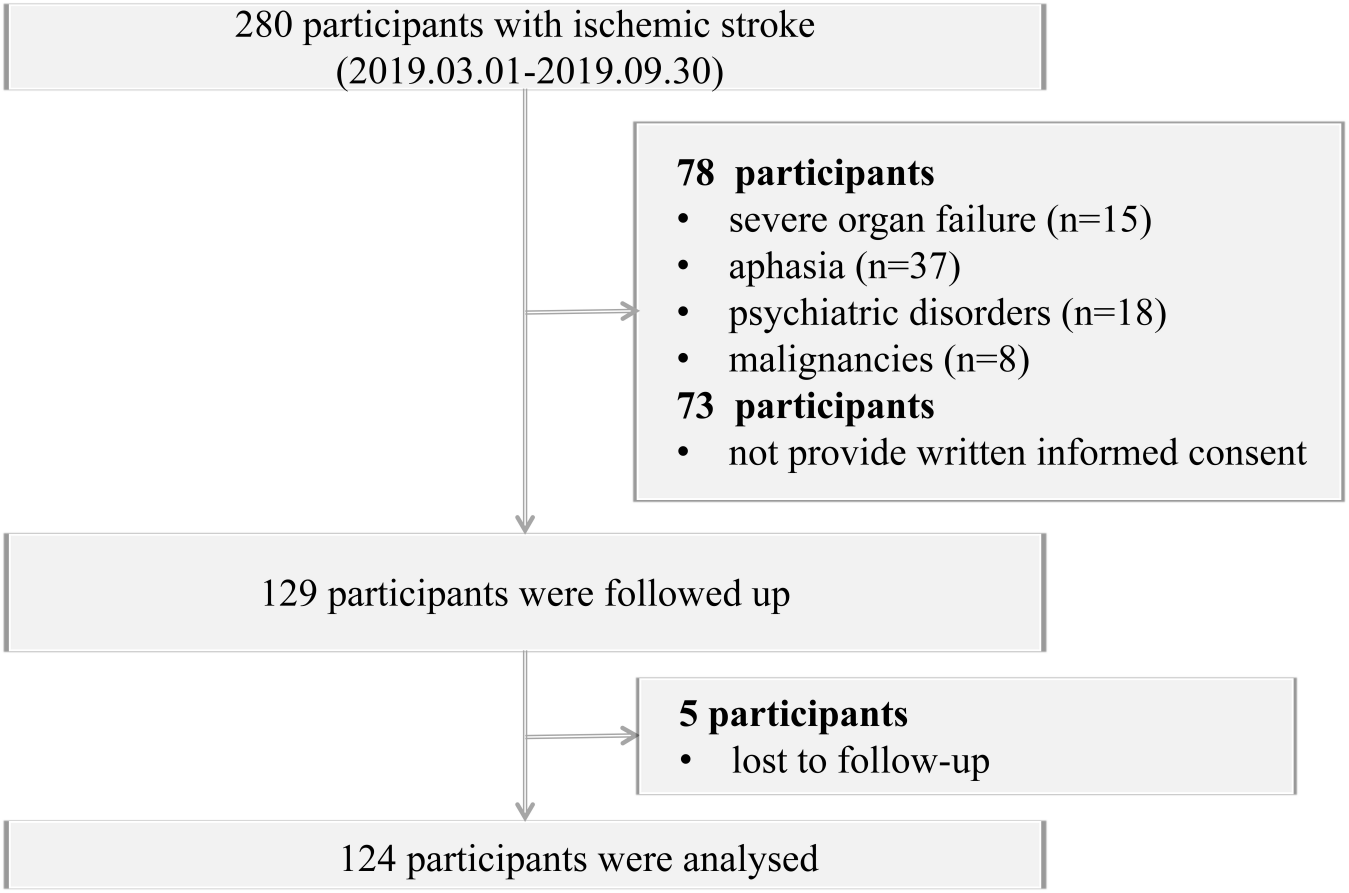
Study flow chat.

Our study initially screened 280 patients with ischemic stroke admitted to the Department of Neurology at the Second People’s Hospital of Neijiang from May 1, 2019, to September 30, 2019. According to the guidelines (29), acute ischemic stroke was defined as: (1) acute onset; (2) presence of focal neurological deficits (e.g., unilateral facial or limb weakness or numbness, aphasia, etc.); (3) identification of a responsible lesion on CT/MRI or persistence of symptoms/signs for >24 h; (4) exclusion of non-vascular etiologies; and (5) exclusion of intracerebral hemorrhage on imaging. Among all patients, we excluded patients (1) with severe organ failure (*n* = 13), aphasia [Boston Diagnostic Aphasia Battery grade 0–2 (30), *n* = 25], psychiatric disorders (*n* = 34), or malignancies (*n* = 6); (2) lacked full cognitive capacity or refused to sign the informed consent form (*n* = 73); (3) who were lost to follow-up (*n* = 5). Finally, 124 patients with acute ischemic stroke were included in the study (Figure 1). This study was supported by the China National Stroke Screening Survey (CNSSS). It was approved by the Medical Ethics Committee of the Second People’s Hospital of Neijiang, with approval number 2018073. All participants and their proxies provided written informed consent.

Prior to the implementation of the study, all data collectors underwent standardized training, which included structured questionnaire interviews, standardized blood pressure measurement, and standardized laboratory testing procedures. Only those who passed the assessment were permitted to participate in the study. An independent quality control group was established during the study to dynamically monitor the entire data collection process, including questionnaire completion, physical examination, and laboratory testing. Participants were admitted within 72 h of symptom onset and all baseline data were collected within 48 h of hospital admission. Data management was conducted using a dual-independent data entry system. After data entry, consistency checks were performed. Any discrepancies in the data entries were corrected after dual verification with the original paper records.

### Family support assessment

Family support was assessed using the Family Support Questionnaire (FSQ) and the Family Adaptation, Partnership, Growth, Affection and Resolve (APGAR) Questionnaire. The FSQ is an appropriate instrument for assessing family support in the Chinese population and has been validated in previous study (Cronbach’s α = 0.85) (31), with reference to the Family Support Scale (Perceived Social Support from Family Scale, PSS-Fa) designed by Procidano and Heller in the United States in 1983 (32–34). The FSQ captures tangible supportive behaviors (emotion, finance, daily affairs, or health) directly relevant to post-stroke recovery (35). The total score ranges from 0 to 15 points, based on the scores, family behavioral support is categorized into three levels: low level (0–5 points), medium level (6–10 points), and high level (11–15 points).

The Family APGAR Questionnaire was developed by American scholar Smilkstein in 1978 (36). The APGAR Questionnaire has been validated and shown to be appropriate for use in the Chinese population, a survey conducted among 2,635 Chinese individuals demonstrated that the APGAR Questionnaire has good internal consistency in the Chinese population (Cronbach’s α = 0.94) (37). The Family APGAR Questionnaire captures the close relationships between the respondents and other family members, it is widely used in stroke recovery (38, 39). The total score ranges from 0 to 10 points. Based on the scores, family function is categorized into three levels: severe dysfunction (0–3 points), moderate dysfunction (4–6 points), and good function (7–10 points), which correspond to low, medium, and high levels of family care and support, respectively.

### Outcome assessment

The primary outcome was assessed using the internationally recommended scale for functional evaluation of acute ischemic stroke (Chinese version), namely the Modified Rankin Scale (mRS) (40–42). Previously, several studies in China used mRS to assess the functional status of stroke patients (42–44). The mRS score ranges from 0 to 6, divided into seven levels. Based on previous studies, we defined scores of 0–2 as functional independence and scores of 3–6 as functional dependence (45, 46). We evaluated the mRS scores of participants both at admission and 6 months after discharge. Additionally, we calculated the difference between the mRS score at 6 months after discharge and the admission score, denoted as ΔmRS.

Secondary outcomes included changes in lifestyle behaviors (alcohol consumption, smoking, exercise, and healthy diet score) and medication adherence at follow-up, which are key modifiable risk factors in clinical guidelines. Alcohol consumption, smoking status and physical activity were assessed with standardized instruments that have been validated (47–50). The healthy diet score (51) was assessed by seven components, details of which can be referred to in previous studies. Medication adherence was evaluated using the Brief Medication Questionnaire (BMQ) (52), which includes necessity beliefs and concern beliefs. The difference between these two beliefs constitutes the final score. The BMQ has been validated in the Chinese population and has demonstrated good validity and reliability (Cronbach’s α = 0.759) (53).

### Covariates assessment

The following covariates were assessed: age (years), sex (male/female), educational status (years of schooling: <6, 6–9, >9), alcohol status (current, former/never), smoking status (current, former/never), physical activity (whether meeting the guideline recommendations) (54), hypertension (yes/no), diabetes (yes/no), hyperlipidemia (yes/no), and National Institutes of Health Stroke Scale (NIHSS) score. The above information was obtained from patient self-reports or electronic medical records during hospitalization.

### Statistical analysis

Continuous data are presented as mean and standard deviation. Categorical data are described using frequencies and percentages. For functional recovery, univariate and multivariate logistic regression analyses were built. With low family support participants as the reference group, the multivariate logistic regression analysis was adjusted for all the covariates mentioned above and baseline mRS scores. Additionally, changes in mRS scores were assessed using univariate and multivariate linear regression, with the same adjustments as in the multivariate logistic regression. For secondary outcomes, ANOVA analysis and chi-square tests were used according to the data types. Statistical significance was defined as a *p*-value < 0.05 for two-tailed tests. All analyses were performed using SPSS version 27.0 (IBM Corp., Armonk, NY, USA).

## Results

### Baseline characteristics

A total of 124 participants were included in this study (age: 68.44 ± 11.27 years, male: 71 [57.26%]) with follow-up of 6 months. At baseline, there were no significant differences among participants with different levels of FSQ and APGAR score in terms of age, gender ratio, smoking and alcohol history, hypertension, hyperglycemia, hyperlipidemia, and mRS scores (Table 1).

**TABLE 1:** Participants characteristics at baseline.

Variables	Overall	FSQ score			*P*	APGAR score			*P*
		Low	Medium	High		Low	Medium	High	
Sample size, *n* (%)	124	24	45	55		30	33	61	
Sex, *n* (%)					0.053				0.646
	71 (57.26)	15 (62.50)	31 (68.89)	25 (45.45)		15 (50.00)	20 (60.61)	36 (59.02)	
	53 (42.74)	9 (37.50)	14 (31.11)	30 (54.55)		15 (50.00)	13 (39.39)	25 (40.98)	
Age (years)	68.44 (11.27)	70.17 (12.92)	66.71 (12.07)	69.09 (9.77)	0.409	69.67 (11.66)	70.36 (11.86)	66.79 (10.68)	0.271
Education, *n* (%)					0.843				0.193
<6 years	76 (61.29)	15 (62.50)	28 (62.22)	33 (60.00)		12 (40.00)	24 (72.73)	40 (65.57)	
6–9 years	31 (25.00)	7 (29.17)	10 (22.22)	14 (25.45)		12 (40.00)	6 (18.18)	13 (21.31)	
>9 years	10 (8.06)	2 (8.33)	3 (6.67)	5 (9.09)		4 (13.33)	2 (6.06)	4 (6.56)	
Other	7 (5.65)	0 (0.00)	4 (8.89)	3 (5.45)		2 (6.67)	1 (3.03)	4 (6.56)	
Hypertension	84 (67.74)	18 (75.00)	28 (62.22)	38 (69.09)	0.535	21 (70.00)	22 (66.67)	41 (67.21)	0.953
Diabetes, *n* (%)	47 (35.48)	8 (29.17)	18 (37.78)	21 (36.36)	0.931	14 (43.33)	14 (42.42)	19 (27.87)	0.408
Hyperlipidemia, *n* (%)	21 (16.94)	3 (12.50)	10 (22.22)	8 (14.55)	0.484	5 (16.67)	4 (12.12)	12 (19.67)	0.647
Current smoking, *n* (%)	34 (27.42)	7 (29.17)	15 (33.33)	12 (21.82)	0.429	7 (23.33)	9 (27.27)	18 (29.51)	0.825
Current drinking, *n* (%)	26 (20.97)	6 (25.00)	13 (28.89)	7 (12.73)	0.123	6 (20.00)	11 (33.33)	9 (14.75)	0.106
Physical activity	8 (6.45)	2 (8.33)	3 (6.67)	3 (5.45)	0.889	2 (6.67)	2 (6.06)	4 (6.56)	0.994
Healthy diet score	2.44 (1.18)	2.96 (1.12)	2.38 (1.09)	2.27 (1.24)	0.053	2.80 (1.03)	2.67 (1.29)	2.15 (1.14)	0.020
NIHSS score	4.18 (4.34)	4.88 (3.98)	3.20 (4.33)	4.67 (4.43)	0.164	5.30 (5.77)	3.88 (2.80)	3.79 (4.19)	0.267
mRS score at baseline	2.02 (1.29)	1.71 (1.04)	2.31 (1.44)	1.93 (1.23)	0.137	2.13 (1.38)	1.82 (1.21)	2.08 (1.29)	0.559

FSQ, Family Support Questionnaire; NIHSS, National Institutes of Health Stroke Scale; mRS, Modified Rankin Scale.

### Association between FSQ scores and mRS scores

**FIGURE 2:**
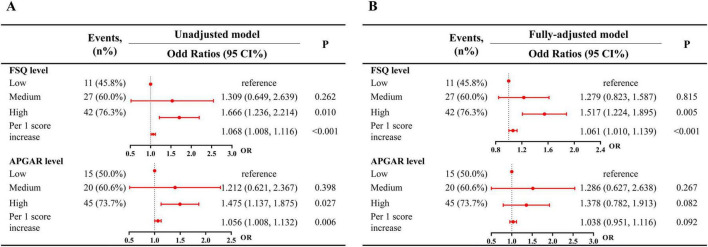
Association between family support level and functional independence. **(A)** Unadjusted model; **(B)** fully-adjusted models. Fully-adjusted models were adjusted for age, sex, educational status, alcohol status, smoking status, physical activity, hypertension, diabetes, hyperlipidemia, and NIHSS score.

In the unadjusted model (Figure 2), compared with low-support patients, those with high support exhibited a 52% relative increase in the odds of functional independence (OR = 1.666, 95% CI 1.236–2.214, *P* = 0.010). Additionally, each one-point increase in the FSQ score was associated with a 6.8% increase in the odds of functional independence (OR = 1.068, 95% CI 1.008–1.116, *P* < 0.001). In the fully adjusted logistic regression (Figure 2), participants with high FSQ score level had an odds ratio (OR) of 1.517 (95% CI 1.224–1.895, *P* = 0.005), and each one-point increase was associated with an OR of 1.061 (95% CI 1.004–1.129, *P* < 0.001).

In the fully adjusted linear regression (Table 2), in the high FSQ score group, mRS scores decreased (β = −1.001, 95% CI −1.418 to −0.584, *P* < 0.001) compared to low FSQ score group and each one-point increase in FSQ score was associated with a mean decrease in mRS scores of 0.127 (β = −0.127, 95% CI −0.179 to −0.075, *P* < 0.001). Thus, higher FSQ score was linked to greater functional independence in post-stroke patients.

**TABLE 2:** Association between family support level and ΔmRS score.

Variables	Unadjusted model		Fully-adjusted model	
	β (95% CI)	*P*	β (95% CI)	*P*
**FSQ level**				
Low	Reference		Reference	
Medium	−0.975 (−1.787, −0.163)	0.019	−0.164 (−0.639, 0.311)	0.492
High	−1.096 (−1.832, −0.360)	0.004	−1.001 (−1.418, −0.584)	<0.001
Per 1 score increase	−0.146 (−0.223, −0.068)	<0.001	−0.127 (−0.179, −0.075)	<0.001
**APGAR level**				
Low	Reference		Reference	
Medium	−0.206 (−0.603, 1.016)	0.613	−0.098 (−0.671, 0.474)	0.731
High	−0.515 (−1.244, 0.215)	0.165	−0.302 (−0.725, 0.122)	0.160
Per 1 score increase	−0.120 (−0.232, −0.008)	0.036	−0.073 (−0.138, −0.007)	0.030

Fully-adjusted model were adjusted for age, sex, educational status, alcohol status, smoking status, physical activity, hypertension, diabetes, hyperlipidemia, and NIHSS score.

### Association between APGAR scores and mRS scores

In the unadjusted model (Figure 2), compared to low score groups, high APGAR score group were associated with greater functional independence (OR = 1.475, 95% CI 1.137–1.875, *P* = 0.027). For each one-point increase in APGAR scores, the odds of functional independence increased by 5.6% (OR = 1.056, 95% CI 1.008–1.132, *P* = 0.006). However, in the fully adjusted logistic regression (Figure 2), APGAR score level was not significantly associated with functional independence (*P* > 0.05).

In the fully adjusted linear regression (Table 2), there was no association between APGAR score group and mRS scores decreased. While each one-point increase in APGAR score was associated with a mean decrease in mRS scores of 0.073 (β = −0.073, 95% CI −0.138 to −0.007, *P* = 0.030). Although APGAR categorical variable was no longer significantly associated with functional independence after multivariable adjustment, each unit increase in its continuous score remained associated with measurable improvement in mRS.

### Association between lifestyle behaviors and medication adherence

Compared to the baseline, the overall proportion of current smokers (27.42% vs. 12.10%) and drinkers (20.97% vs. 5.65%) among participants was reduced after 6 months, while the healthy diet score (2.44 ± 1.18 vs. 3.91 ± 0.71) and proportion of exercising (6.45% vs. 20.97%) increased. However, there were no significant differences in smoking, drinking, exercise, and healthy diet scores among the groups with different levels of FSQ and APGAR scores (Table 3). In terms of medication adherence (Table 3), a higher level of FSQ score (low vs. medium vs. High: 3.24 ± 1.48 vs. 3.73 ± 1.03 vs. 5.40 ± 1.01, *P* < 0.001) and APGAR score (low vs. medium vs. high: 4.30 ± 1.16 vs. 4.33 ± 1.51 vs. 4.76 ± 1.47, *P* = 0.039) were associated with higher medication adherence. Collectively, higher levels of FSQ and APGAR scores were statistically associated with better medication adherence but not with short-term lifestyle change.

**TABLE 3:** Analyses to lifestyle and medication adherence after 6 months.

Variables	Overall	FSQ score			*P*	APGAR score			*P*
		Low	Medium	High		Low	Medium	High	
**Current smoking, *n* (%)**									
No	109 (87.90)	23 (95.83)	36 (80.00)	50 (90.91)	0.104	27 (90.00)	30 (90.91)	52 (85.25)	0.667
Yes	15 (12.10)	1 (4.17)	9 (20.00)	5 (9.09)		3 (10.00)	3 (9.09)	9 (14.75)	
**Current drinking, *n* (%)**									
No	117 (94.35)	23 (95.83)	41 (91.11)	53 (96.36)	0.496	28 (93.33)	29 (87.88)	60 (98.36)	0.106
Yes	7 (5.65)	1 (4.17)	4 (8.89)	2 (3.64)		2 (6.67)	4 (12.12)	1 (1.64)	
**Physical activity**									
Yes	98 (79.03)	23 (95.83)	33 (73.33)	42 (76.36)	0.074	26 (86.67)	29 (87.88)	43 (70.49)	0.071
No	26 (20.97)	1 (4.17)	12 (26.67)	13 (23.64)		4 (13.33)	4 (12.12)	18 (29.51)	
Healthy diet score	3.91 (0.71)	3.88 (0.45)	4.04 (0.71)	3.82 (0.80)	0.276	3.93 (0.69)	3.88 (0.65)	3.92 (0.76)	0.950
BMQ score	4.40 (1.44)	3.24 (1.48)	3.73 (1.03)	5.40 (1.01)	<0.001	4.30 (1.16)	4.33 (1.51)	4.76 (1.47)	0.039

FSQ, Family Support Questionnaire; BMQ, Brief Medication Questionnaire.

## Discussion

In this study, 124 patients with acute ischemic stroke were included, with an average age of 68 years, mainly distributed in the age group of 60–69 years, which is consistent with the age characteristics of the stroke registry data in China (55). High level of family support (FSQ 11–15 points) showed 1.52-fold higher odds of functional independence and each one-point increase in the FSQ was associated with a 0.127-point decrease in the mRS score, extending previous reports that robust family support improves functional recovery after stroke by quantifying the dose–response relationship. In contrast, APGAR score levels were no longer associated with functional independence after multivariable adjustment. Regardless of whether assessed by FSQ or APGAR score, higher family support was associated with better medication adherence. It should be noted that, although we found that the overall lifestyle improved after stroke compared with before, no significant differences were found between different levels of family support. In summary, this study further highlights the importance of family support for post-stroke functional recovery.

Family support refers to the psychological, social, and behavioral support provided by family members to patients. It has a positive impact on patients’ prognosis, including depression, anxiety, relationship satisfaction, disability, and mortality after illness (56, 57). The Family Support Questionnaire (FSQ) score is mainly used to assess the degree of family support perceived by individuals, quantifying the level of support provided by family members in terms of emotion, finance, daily affairs, or health (35). High levels of family support may be associated with improved patient prognosis. For example, a study in Iran targeting the elderly in rural communities showed that high level of family support were significantly associated with increased activities of daily living among the elderly (58). Additionally, a cross-sectional study demonstrated a positive correlation between high levels of family support and self-efficacy in stroke patients (29). However, an Indonesian tuberculosis cohort found no association between family support and anti-tuberculosis medication adherence (59).

The APGAR score primarily focuses on family function (60). Previous studies showed that lower APGAR score may be an important factor in the occurrence of post-stroke fatigue and lower health beliefs among stroke patients (61, 62). Additionally, the APGAR score was not significantly correlated with patient gender, age, marital status, education level, or socioeconomic status (63). In this study, after fully adjusting the model, the APGAR score was not associated with functional recovery of patients with acute ischemic stroke, which may suggest that family support assessed by FSQ score may play a more important role in the functional recovery of stroke patients and further studies are needed to explore the underlying reasons.

Due to the long recovery time for stroke patients, high hospitalization costs and lack of medical resources, most stroke patients in China only conduct rehabilitation exercise at home after discharge (28, 64, 65). Therefore, functional recovery supported by the family is particularly important for stroke patients in China. Our study clarified the positive impact of family support level on the recovery of patients with acute ischemic stroke, offering a new perspective for improving stroke functional recovery in areas with limited medical resources. This may be related to better family support increasing patients’ health beliefs, self-efficacy, emotional improvement, and medication adherence. Although our study found that lifestyle behaviors were similar in different levels of FSQ and APGAR scores at both baseline and the 6-months follow-up, this may be due to the short follow-up period, which led to insufficient observation.

The study has several limitations. First, the observational, non-randomized design precludes causal inference; all reported associations should be interpreted as correlational. Second, neither patients nor outcome assessors were blinded to the support-level grouping, introducing potential assessor bias in the 6-months mRS evaluation and response bias in self-reported questionnaires. Third, the evaluation of family support and medication adherence relied entirely on patient self-report, which is susceptible to recall and social-desirability bias. Fourth, the single-center design and relatively small sample (*n* = 124) limit external validity and the ability to detect small effect sizes, especially in the lifestyle subgroup analyses. Fifth, the 6-months follow-up, while sufficient for functional outcomes, may be too short to observe the full trajectory of medication adherence or lifestyle change, and longer-term assessments are warranted. Finally, the study did not record the exact relationship between the patient and the family member providing support; thus, we could not examine whether relationship type influences the level of family support. Future studies need to be large-scale and have longer follow-up periods to clarify the impact of family support level on the prognosis of stroke patients.

## Conclusion

The level of family support is positively correlated with functional recovery, and improvement of medication adherence may be a potential mechanism. Our study suggests that family support needs to be assessed among stroke patients after discharge. Enhancing family support may be an essential pathway to improve functional recovery of stroke patients in limited medical resources areas. Future research should investigate the association between family support levels and lifestyle improvements with larger samples and longer follow-up periods.

## Data Availability

The original contributions presented in this study are included in this article/supplementary material, further inquiries can be directed to the corresponding author.

## References

[1] Murray CJL. The global burden of disease study at 30 years. *Nat Med.* (2022) 28:2019–26. 10.1038/s41591-022-01990-1 36216939

[2] Katan M Luft A. Global burden of stroke. *Semin Neurol.* (2018) 38:208–11. 29791947 10.1055/s-0038-1649503

[3] Hou S Zhang Y Xia Y Liu Y Deng X Wang W Global, regional, and national epidemiology of ischemic stroke from 1990 to 2021. *Eur J Neurol.* (2024) 31:e16481. 10.1111/ene.16481 39290044 PMC11555022

[4] Donkor ES. Stroke in the 21(st) Century: a snapshot of the burden, epidemiology, and quality of life. *Stroke Res Treat.* (2018) 2018:3238165. 10.1155/2018/3238165 30598741 PMC6288566

[5] Mendis S. Stroke disability and rehabilitation of stroke: World Health Organization perspective. *Int J Stroke.* (2013) 8:3–4. 23280261 10.1111/j.1747-4949.2012.00969.x

[6] Powers WJ Rabinstein AA Ackerson T Adeoye OM Bambakidis NC Becker K Guidelines for the Early Management of Patients With Acute Ischemic Stroke: 2019 Update to the 2018 Guidelines for the Early Management of Acute Ischemic Stroke: A Guideline for Healthcare Professionals From the American Heart Association/American Stroke Association. *Stroke.* (2019) 50:e344–418. 10.1161/str.0000000000000211 31662037

[7] Pierot L Jarayaman M Szikora I Hirsch J Baxter B Miyachi S Standards of practice in acute ischemic stroke intervention international recommendations. *Can J Neurol Sci.* (2019) 46:269–74. 10.1017/cjn.2019.1 30890199

[8] Nogueira RG Jadhav AP Haussen DC Bonafe A Budzik RF Bhuva P Thrombectomy 6 to 24 hours after stroke with a mismatch between deficit and infarct. *N Engl J Med.* (2018) 378:11–21. 10.1056/NEJMoa1706442 29129157

[9] Leng T Xiong ZG. Treatment for ischemic stroke: From thrombolysis to thrombectomy and remaining challenges. *Brain Circ.* (2019) 5:8–11. 10.4103/bc.bc_36_18 31001594 PMC6458775

[10] The National Institute of Neurological Disorders and Stroke (Ninds) rt-Pa Stroke Study Group. A systems approach to immediate evaluation and management of hyperacute stroke: Experience at eight centers and implications for community practice and patient care. *Stroke.* (1997) 28:1530–40. 10.1161/01.str.28.8.1530 9259745

[11] Knoflach M Matosevic B Rücker M Furtner M Mair A Wille G Functional recovery after ischemic stroke–a matter of age: data from the Austrian Stroke Unit Registry. *Neurology.* (2012) 78:279–85. 10.1212/WNL.0b013e31824367ab 22238419

[12] Levasseur MA Ferrari M McIlwaine S Iyer SN. Peer-driven family support services in the context of first-episode psychosis: Participant perceptions from a Canadian early intervention programme. *Early Interv Psychiatry.* (2019) 13:335–41. 10.1111/eip.12771 30548396

[13] Seshadri K Sivakumar T Jagannathan A. The Family Support Movement and Schizophrenia in India. *Curr Psychiatry Rep.* (2019) 21:95. 10.1007/s11920-019-1081-5 31522258

[14] Smith LN Lawrence M Kerr SM Langhorne P Lees KR. Informal carers’ experience of caring for stroke survivors. *J Adv Nurs.* (2004) 46:235–44. 10.1111/j.1365-2648.2004.02983.x 15066101

[15] Barskova T Wilz G. Interdependence of stroke survivors’ recovery and their relatives’ attitudes and health: a contribution to investigating the causal effects. *Disabil Rehabil.* (2007) 29:1481–91. 10.1080/09638280601029399 17882729

[16] Dennis M O’Rourke S Slattery J Staniforth T Warlow C. Evaluation of a stroke family care worker: results of a randomised controlled trial. *Bmj.* (1997) 314:1071–6; discussion 1076–7. 10.1136/bmj.314.7087.1071 9133884 PMC2126479

[17] Mant J Winner S Roche J Wade DT. Family support for stroke: one year follow up of a randomised controlled trial. *J Neurol Neurosurg Psychiatry.* (2005) 76:1006–8. 10.1136/jnnp.2004.048991 15965213 PMC1739696

[18] Seifi A Elliott RJ Elsehety MA. Impact of Patients’ income on stroke prognosis. *J Stroke Cerebrovasc Dis.* (2016) 25:2308–11. 10.1016/j.jstrokecerebrovasdis.2016.05.024 27266622

[19] Khan F Turner-Stokes L Ng L Kilpatrick T. Multidisciplinary rehabilitation for adults with multiple sclerosis. *Postgrad Med J.* (2008) 84:385. 10.1136/jnnp.2007.127563 18716020

[20] Determeijer JJ van Waard JD Leopold SJ Spijker R Agyemang C Vugt MV. The barriers and facilitators family caregivers experience when participating in resource-limited hospital care: a qualitative systematic review. *BMJ Glob Health.* (2024) 9:e015956. 10.1136/bmjgh-2024-015956 39537388 PMC11575306

[21] Holmes AM Deb P. The effect of chronic illness on the psychological health of family members. *J Ment Health Policy Econ.* (2003) 6:13–22. 14578544

[22] Ma Q Li R Wang L Yin P Wang Y Yan C Temporal trend and attributable risk factors of stroke burden in China, 1990-2019: an analysis for the Global Burden of Disease Study 2019. *Lancet Public Health.* (2021) 6:e897–906. 10.1016/s2468-2667(21)00228-0 34838196 PMC9047702

[23] Wu S Wu B Liu M Chen Z Wang W Anderson CS Stroke in China: advances and challenges in epidemiology, prevention, and management. *Lancet Neurol.* (2019) 18:394–405. 10.1016/s1474-4422(18)30500-3 30878104

[24] Bernhardt J Hayward KS Kwakkel G Ward NS Wolf SL Borschmann K Agreed definitions and a shared vision for new standards in stroke recovery research: the Stroke Recovery and Rehabilitation Roundtable taskforce. *Int J Stroke.* (2017) 12:444–50. 10.1177/1747493017711816 28697708

[25] Rosbergen IC Grimley RS Hayward KS Walker KC Rowley D Campbell AM Embedding an enriched environment in an acute stroke unit increases activity in people with stroke: a controlled before-after pilot study. *Clin Rehabil.* (2017) 31:1516–28. 10.1177/0269215517705181 28459184

[26] Prabhakaran S Zarahn E Riley C Speizer A Chong JY Lazar RM Inter-individual variability in the capacity for motor recovery after ischemic stroke. *Neurorehabil Neural Repair.* (2008) 22:64–71. 10.1177/1545968307305302 17687024

[27] Stinear CM Smith MC Byblow WD. Prediction tools for stroke rehabilitation. *Stroke.* (2019) 50:3314–22. 10.1161/strokeaha.119.025696 31610763

[28] Wagachchige Muthucumarana M Samarasinghe K Elgán C. Caring for stroke survivors: experiences of family caregivers in Sri Lanka - a qualitative study. *Top Stroke Rehabil.* (2018) 25:397–402. 10.1080/10749357.2018.1481353 30028654

[29] Liu L Chen W Zhou H Duan W Li S Huo X Chinese Stroke Association guidelines for clinical management of cerebrovascular disorders: executive summary and 2019 update of clinical management of ischaemic cerebrovascular diseases. *Stroke Vasc Neurol.* (2020) 5:159–76. 10.1136/svn-2020-000378 32561535 PMC7337371

[30] Draper I. The assessment of aphasia and related disorders. *J Neurol Neurosurg Psychiatry.* (1973) 36:894.

[31] Zhang HY. Correlation between family support and self-care behaviors in breast cancer patients. *J Nurs Sci.* (1999) 14:195–6.

[32] Li G Hu H Dong Z Arao T. Development of the Chinese family support scale in a sample of Chinese patients with hypertension. *PLoS One.* (2013) 8:e85682. 10.1371/journal.pone.0085682 24376892 PMC3869941

[33] Procidano ME Heller K. Measures of perceived social support from friends and from family: three validation studies. *Am J Community Psychol.* (1983) 11:1–24. 10.1007/bf00898416 6837532

[34] Yang X Xue M Pauen S He H. Psychometric properties of the chinese version of multidimensional scale of perceived social support. *Psychol Res Behav Manag.* (2024) 17:2233–41. 10.2147/prbm.S463245 38835653 PMC11149633

[35] Toledano-Toledano F Luna D. The psychosocial profile of family caregivers of children with chronic diseases: a cross-sectional study. *Biopsychosoc Med.* (2020) 14:29. 10.1186/s13030-020-00201-y 33110443 PMC7583305

[36] Smilkstein G. The family APGAR: a proposal for a family function test and its use by physicians. *J Fam Pract.* (1978) 6:1231–9. 660126

[37] Nan H Ni MY Lee PH Tam WW Lam TH Leung GM Psychometric evaluation of the Chinese version of the Subjective Happiness Scale: evidence from the Hong Kong FAMILY Cohort. *Int J Behav Med.* (2014) 21:646–52. 10.1007/s12529-014-9389-3 24515396 PMC4107280

[38] de Oliveira SC dos Santos AA Pavarini SC. [The relationship between depressive symptoms and family functioning in institutionalized elderly]. *Rev Esc Enferm USP.* (2014) 48:66–72. 10.1590/s0080-623420140000100008 24676110

[39] Zhang W Gao YJ Ye MM Zhou LS. Post-stroke family resilience is correlated with family functioning among stroke survivors: The mediating role of patient’s coping and self-efficacy. *Nurs Open.* (2024) 11:e2230. 10.1002/nop2.2230 38940513 PMC11212063

[40] Broderick JP Adeoye O Elm J. Evolution of the modified rankin scale and its use in future stroke trials. *Stroke.* (2017) 48:2007–12. 10.1161/strokeaha.117.017866 28626052 PMC5552200

[41] van Swieten JC Koudstaal PJ Visser MC Schouten HJ van Gijn J. Interobserver agreement for the assessment of handicap in stroke patients. *Stroke.* (1988) 19:604–7. 10.1161/01.str.19.5.604 3363593

[42] Yuan JL Bruno A Li T Li SJ Zhang XD Li HY Replication and extension of the simplified modified rankin scale in 150 Chinese stroke patients. *Eur Neurol.* (2012) 67:206–10. 10.1159/000334849 22377778

[43] Tao C Liu T Cui T Liu J Li Z Ren Y Early tirofiban infusion after intravenous thrombolysis for stroke. *N Engl J Med.* (2025) 393:1191–201. 10.1056/NEJMoa2503678 40616232

[44] Yuan J Wang Y Hu W Bruno A. The reliability and validity of a novel Chinese version simplified modified Rankin scale questionnaire (2011). *BMC Neurol.* (2020) 20:127. 10.1186/s12883-020-01708-1 32268886 PMC7140377

[45] Krieger P Melmed KR Torres J Zhao A Croll L Irvine H Pre-admission antithrombotic use is associated with 3-month mRS score after thrombectomy for acute ischemic stroke. *J Thromb Thrombolysis.* (2022) 54:350–9. 10.1007/s11239-022-02680-y 35864280 PMC9302951

[46] Albers GW Marks MP Kemp S Christensen S Tsai JP Ortega-Gutierrez S Thrombectomy for Stroke at 6 to 16 hours with selection by perfusion imaging. *N Engl J Med.* (2018) 378:708–18. 10.1056/NEJMoa1713973 29364767 PMC6590673

[47] Wang YJ Li ZX Gu HQ Zhai Y Zhou Q Jiang Y China Stroke Statistics: an update on the 2019 report from the National Center for Healthcare Quality Management in Neurological Diseases, China National Clinical Research Center for Neurological Diseases, the Chinese Stroke Association, National Center for Chronic and Non-communicable Disease Control and Prevention, Chinese Center for Disease Control and Prevention and Institute for Global Neuroscience and Stroke Collaborations. *Stroke Vasc Neurol.* (2022) 7:415–50. 10.1136/svn-2021-001374 35443985 PMC9614174

[48] Craig CL Marshall AL Sjöström M Bauman AE Booth ML Ainsworth BE International physical activity questionnaire: 12-country reliability and validity. *Med Sci Sports Exerc.* (2003) 35:1381–95. 10.1249/01.Mss.0000078924.61453.Fb 12900694

[49] Yang GH Li Q Wang CX Hsia J Yang Y Xiao L Findings from 2010 Global Adult Tobacco Survey: implementation of MPOWER policy in China. *Biomed Environ Sci.* (2010) 23:422–9. 10.1016/s0895-3988(11)60002-0 21315239

[50] Claussen B Aasland OG. The Alcohol Use Disorders Identification Test (AUDIT) in a routine health examination of long-term unemployed. *Addiction.* (1993) 88:363–8. 10.1111/j.1360-0443.1993.tb00823.x 8461853

[51] Liu W Wang T Zhu M Jin G. Healthy diet, polygenic risk score, and upper gastrointestinal cancer risk: a prospective study from UK Biobank. *Nutrients.* (2023) 15:1344. 10.3390/nu15061344 36986074 PMC10054787

[52] Krass I Taylor SJ Smith C Armour CL. Impact on medication use and adherence of Australian pharmacists’ diabetes care services. *J Am Pharm Assoc.* (2003) 45:33–40. 10.1331/1544345052843093 15730115

[53] Cai Q Ye L Horne R Bi J Xu Q Ye X Patients’ adherence-related beliefs about inhaled steroids: application of the Chinese version of the Beliefs about Medicines Questionnaire-specific in patients with asthma. *J Asthma.* (2020) 57:319–26. 10.1080/02770903.2019.1565824 30663909

[54] Billinger SA Arena R Bernhardt J Eng JJ Franklin BA Johnson CM Physical activity and exercise recommendations for stroke survivors: a statement for healthcare professionals from the American Heart Association/American Stroke Association. *Stroke.* (2014) 45:2532–53. 10.1161/str.0000000000000022 24846875

[55] Wang W Jiang B Sun H Ru X Sun D Wang L Prevalence, incidence, and mortality of stroke in China: results from a nationwide population-based survey of 480 687 Adults. *Circulation.* (2017) 135:759–71. 10.1161/circulationaha.116.025250 28052979

[56] Martire LM Lustig AP Schulz R Miller GE Helgeson VS. Is it beneficial to involve a family member? A meta-analysis of psychosocial interventions for chronic illness. *Health Psychol.* (2004) 23:599–611. 10.1037/0278-6133.23.6.599 15546228

[57] Fiacco S Mernone L Ehlert U. Psychobiological indicators of the subjectively experienced health status - findings from the Women 40+ Healthy Aging Study. *BMC Womens Health.* (2020) 20:16. 10.1186/s12905-020-0888-x 31996204 PMC6988289

[58] Jokar F Asadollahi AR Kaveh OH Ghahramani L Nazari M. Relationship of perceived social support with the activities of daily living in older adults living in rural communities in Iran. *Salmand-Iranian J Ageing.* (2020) 15:350–64. 10.32598/sija.10.15.3.2773.2

[59] Yani DI Juniarti N Lukman M. Factors related to complying with Anti-TB medications among drug-resistant tuberculosis patients in Indonesia. *Patient Prefer Adherence.* (2022) 16:3319–27. 10.2147/ppa.S388989 36568917 PMC9769133

[60] Koch Filho HR Koch LFA Kusma SZ Ignácio SA Moysés ST Alanis LRA Self-perception of gerontoism according to social support and family functionality. *Iran J Public Health.* (2019) 48:673–80. 31110977 PMC6500520

[61] Zhang L Shu Y Han C Liu J. Correlation between family functioning and health beliefs in patients with stroke in Beijing, China. *J Multidiscip Healthc.* (2023) 16:1067–74. 10.2147/jmdh.S394396 37096237 PMC10122470

[62] Zhu R Huang H Yu Y Bao S Lin N Shu M. Post-stroke fatigue and its correlation with family functioning in patients who have experienced a first episode of stroke. *Front Aging Neurosci.* (2024) 16:1440163. 10.3389/fnagi.2024.1440163 39497785 PMC11532170

[63] Stoll WD Taber DJ Palesch SJ Hebbar L. Utility of the surgical apgar score in kidney transplantation: is it feasible to predict ICU admission, hospital readmission, length of stay, and cost in this patient population? *Prog Transplant.* (2016) 26:122–8. 10.1177/1526924816640948 27207400

[64] Yin Z Deng Y Li Z Gu H Zhou Q Wang Y Assessment of rehabilitation following acute ischaemic stroke in China: a registry-based retrospective observational study. *BMJ Open.* (2024) 14:e082279. 10.1136/bmjopen-2023-082279 38553086 PMC10982726

[65] Zeng S Wu M Xu L Guo Z Chen S Ling K Challenges in accessing community-based rehabilitation and long-term care for older adult stroke survivors and their caregivers: a qualitative study. *J Multidiscip Healthc.* (2024) 17:4829–38. 10.2147/jmdh.S476993 39464787 PMC11512762

